# Influence factors of dental anxiety in patients with impacted third molar extractions and its correlation with postoperative pain: a prospective study

**DOI:** 10.4317/medoral.23293

**Published:** 2020-10-09

**Authors:** Ji Liang Xu, Rong Xia

**Affiliations:** 1 Department of Stomatology, the Second Hospital of Anhui Medical University, Hefei, People's Republic of China

## Abstract

**Background:**

To explore the prevalence of dental anxiety (DA) in patients with third molar extractions and its influence factors and the correlation between DA levels and postoperative pain.

**Material and Methods:**

A prospective and descriptive clinical study was performed. All patients who underwent the impacted third molar extraction from October 2017 to February 2019 were enrolled. DA levels were assessed by virtue of the modified dental anxiety scale (MDAS) and pain was assessed with a visual analog scale (VAS).

**Results:**

A total of 150 patients were investigated and 136 valid questionnaires were retrieved, with an effective rate of 90.7%. The independent sample t-test and ANOVA results showed that the anxiety level of patients with the third molar extractions was statistically different in gender, teeth extraction experience and self-assessment oral health status. Multiple linear regression analysis with DA as a dependent variable showed that gender and teeth extraction experience were independent factors influencing DA in patients with third molar extractions. Pearson's test showed that there was a significant correlation between DA level in patients and the postoperative pain on the first day (r=0.542, *p*=0.000).

**Conclusions:**

For patients (females, poor oral hygiene and no teeth extraction experience), surgeon should pay more attention to DA of such patients and take measures to reduce the anxiety when removing the third molars. Furthermore, surgeon can recommend oral administration ibuprofen sustained release capsules after surgery.

** Key words:**Dental anxiety, modified dental anxiety scale, visual analogue scale, postoperative pain.

## Introduction

Dental anxiety (DA) refers to the patient's tension, anxiety and fear during the dental treatment process, the behavior of which is characterized by increased sensitivity to pain, reduced tolerance, and even the emerging phenomenon of avoiding or refusing to extract teeth (1,2). The studies of the etiology of DA have found that it is produced by a combination of multiple factors, mainly divided into internal and external factors (3). Internal factors are mainly related to the patient's own psychological state. These patients are often accompanied by persistent anxiety and other symptoms, whose pain threshold may be lower than that of normal people, further to affect the degree of DA. While the external factors are mainly related to the patient's direct or indirect dental treatment experience. The experience of direct dental treatment mainly refers to the related treatment experience that the patients once had unpleasantness or even pain, and the traumatic treatment experience has the greatest impact.

DA is a state of mentality and it is difficult to qualify it. At present, it is mainly evaluated by questionnaire survey. The commonly used DA assessment scales are the Corah's Dental Anxiety Scale (DAS) (4), the Modified Dental Anxiety Scale (MDAS) (5), the State-Trait Anxiety Inventory Scale (STAIS) (6), the Dental Fear Survey (DFS) (7) and the Amsterdam Preoperative Anxiety and Information Scale (APAIS) (8). However, there are differences in the reliability, validity and evaluation items for each scales.

DA had a high incidence rate, and its detection rate was generally high. However, due to the difference of research subject selection and the inconsistency of the used scales, the prevalence of DA in different countries and regions was not the same. The surveys of scholars from various countries have respectively obtained the detection rate of DA: Canada 42.5% (9), Netherlands 22% (10), England 25% (11), and America 20% (12).

Due to anxiety and fear, patients often tend to evade or delay treatment and see a doctor or regular oral examination reluctantly, further to influence the rate of early visits and lead to a decline in oral health condition. Patients with a high degree of DA may have more number of dental caries and missing teeth than normal patients. For clinicians, patients with DA usually have nervous performance when they visit the dentists. They could not cooperate well with dentist for the dental treatments, which increases the treatment difficulty and makes the treatment not smooth. On the other hand, patients with DA may have a higher severity of oral diseases than ordinary patients, and requires more complicated treatments. The prognosis of the sick teeth may be poor, which may worsen the relationship between dentists and patients to some extent (13).

The aim of this study was to explore the prevalence of DA in patients with third molar extractions and its influence factors and the correlation between DA levels and postoperative pain, to provide guidance for oral diagnosis and treatment of DA.

## Material and Methods

- Study sample

From October 2017 to February 2019, the patients who underwent the impacted third molar extractions in the department of stomatology, the Second Hospital of Anhui Medical University, were selected as subjects. This study was approved and licensed by the Ethics Committee of the Second Hospital of Anhui Medical University. All patients signed informed consent. The study was adherent to the STROBE (Strengthening the Reporting of Observational Studies in Epidemiology) statement checklist as seen in Supplementary Materials.

Inclusion criteria: 1) Patients were over 18 years of age and agreed to participate in the study; 2) Patients could complete the scale on their own; 3) Patients should not use anti-anxiety drugs, sedatives or analgesics within 3 days before treatment; 4) Patients presented an ASA score I or II.

Exclusion criteria: Patients were excluded, who had cognitive and visual impairment, and history of mental illness and local anesthetic allergy.

- Rating scales and research indicators

The general questionnaire was used to assess the relationship between the general condition of the patients and DA. It consists of gender, age, marital status, regular oral examination or not, initial visit or not, tooth extraction experience, degree of impacted third molars extraction difficulty (14), postoperative anti-inflammatory methods, self-assessment of oral hygiene, income level, education level, and time of visit.

MDAS was used to assess the severity of DA in patients. There were 5 questions. Each question included 5 items. The total scores varied from 5-25 (15). A patient with an MDAS score of ≥12 was diagnosed with DA while a score of ≥19 was considered high degree DA, that is, dental phobia. MDAS consists of two factors: expected DA and therapeutic DA. Currently, MDAS is an internationally accepted scale for DA.

Visual analogue scale (VAS) was used to evaluate the degree of pain on the first day after impacted third molar extractions. The VAS consists of a 10 cm line with 0 at one end for painlessness and 10 at the other end for the most severe pain.

- Research methods and quality control

The patient received an oral examination and took periapical radiograph or oral pantomography in 30 minutes before treatment. A trained dentist would introduce to patients the type of impacted third molars, the general surgical procedure of extractions, possible complications and precautions. After the patients agreed with the impacted third molar extractions, the patients were assisted to complete a general questionnaire, MDAS and patient informed consent. The questionnaires were collected on site after patients completed the survey, and the integrity of the questionnaire was checked. If errors and omissions were found in the questionnaire, the patients would be promptly assisted to correct or refill. The impacted third molars extractions in all patients were performed by the same physician with more than 5 years of work experience. The treatment process was performed under local anesthesia with 4% articaine plus 1:100,000 epinephrine. After the anesthetic was effective, surgeon checked the position of the teeth, and followed the steps below: separating the gingiva, loosing the teeth, placing the forceps, dislocated the teeth. For complex impacted third molars, the surgeon may use incision and envelope flap (16), a high-speed turbine with 45°angle to remove the bone, and teeth or root separation according to the specific situation. At the end of the procedure, the surgeon checked the integrity of the root and then reset the alveolar bone. For cases of mucosal incision, 4-0 non-absorbable suture (Johnson & Johnson, Shanghai, China) was applied to suture wounds. Gauze tampon was advised for compression and hemostasis. Written postoperative instructions were presented. Patients were given oral administrated amoxicillin and metronidazole for 3-5 days: amoxicillin capsules three times a day, 1 g once; metronidazole Tablets three times a day, 0.4 g once. Patients with mucoperiosteal flap opened and bone removal, were recommended to receive infusion anti-inflammatory treatment at least one day: clindamycin 0.6g twice a day, dexamethasone sodium phosphate injection 5mg, once a day, metronidazole chlorination Sodium 0.5g twice a day. The sutures were removed at 5-7 days postoperation. The patients and the surgeon added WeChat (a Chinese chat tool, similar to FaceBook) and phone number in order to communicate and record the postoperative VAS scores on 1st day.

- Statistical analysis

Statistical analysis was performed on the data using the SPSS 10.0 software package. Independent sample t-test and one-way ANOVA were used to treat the relationship between individual factors and DA. Multiple linear regression analysis was performed on the factors with significant correlations to obtain independent factors for DA in patients with third molar extractions. Pearson's test was used to treat the correlation between DA and postoperative VAS. *P*<0.05 was considered statistically significant.

## Results

A total of 150 patients were investigated and 136 valid questionnaires were retrieved, with an effective rate of 90.7%. There were 39 males and 97 females, aged 18-73 years, with an average age of (32.97±10.42) years. According to MDAS results, the average score was 12.804.01 points, of which 50 (36.8%) scored ≤11 points, ie no anxiety or mild anxiety; 77 cases (56.6%) scored 12-18 points, ie moderate anxiety; 9 cases (6.6%) scored≥19 points, ie severe anxiety. The prevalence of DA (MDAS≥12) was 63.2%.

The independent sample t-test and ANOVA results showed that the anxiety level of patients with the third molar extractions was statistically different in gender, teeth extraction experience and self-assessment oral health status, and there was no statistical difference in other factors, as shown in Table 1. Multiple linear regression analysis with DA as a dependent variable showed that gender and teeth extraction experience were independent factors influencing DA in patients with third molar extractions.

Pearson's test showed that there was a significant correlation between DA level in patients with third molar extractions and the postoperative pain on the first day (r=0.542, *p*=0.000), as shown in Table 2.

**Table 1:**
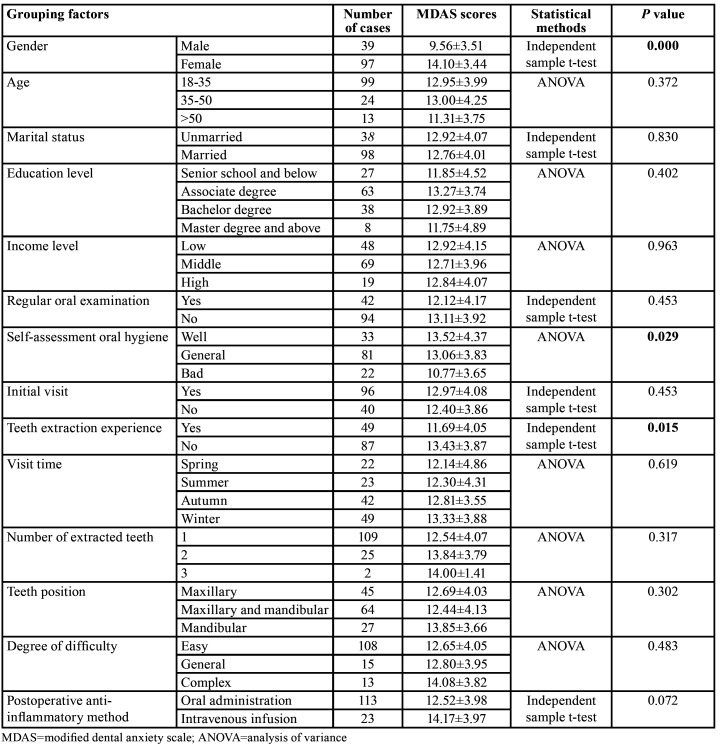
Classification of dental anxiety with different grouping factors.

**Table 2:**
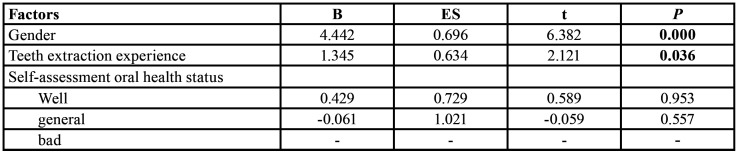
Linear regression results for different grouping factors.

## Discussion

DA has a high incidence rate and has a non-negligible negative impact on the patient's own, clinical work and social influence. Many scholars have conducted long-term researches on DA. Due to the differences in the choice of study subjects and the inconsistency in the use of scales, the prevalence of DA in adult in different countries and regions is also different. Scholars have also indicated that the pathogenesis of DA has certain characteristics. Although many years of research have been conducted, many epidemiological issues regarding adult dental anxiety have not been clearly established, and the prevalence of dental anxiety in different regions is not the same.

In this study, the average score of female MADS was higher than that of males, that is, the degree of female dental anxiety was siginificantly higher than that of males. This result was consistent with many existing studies (17-19). This may be related to the fact that females expressed more fear than males in psychological aspect (17). Currently, it is believed that female's ability to cope with dental visits is significantly lower than that of male, and their desire to control themselves and the weak coping ability in actual dental visits may increase their psychological stress. On the other hand, etiological studies showed that fear of pain was one of the main causes of DA. They generally differ in their perception and control of pain, and females have lower pain thresholds than males (20). The above may cause females to have a higher degree of DA than males. However, there were very few researchers, such as Locker *et al*. (21) who believed that there was no significant correlation between gender and DA. Gender was a common and important factor in current DA research. According to most scholars' research, the DA difference between males and females suggested that female patients were more likely to have DA. The surgeon should need to pay more attention to the reaction of female patients in the process of communication and treatment and take corresponding measures to reduce the degree of anxiety.

In addition, we also found that the average score of MADS in patients with teeth extraction experience was significantly lower than that in patients without. We suspected that this may be attributed to patients, who had previous teeth extraction experience, did more detailed understanding of the extraction process and made more adequate preparation for pain tolerance.

Furthermore, the results of this study showed that the degree of pain on the first day after teeth extractions was positively correlated with the degree of DA, which was consistent with previous studies (22,23). The reason may be related to the psychological preparation for pain in these patients. Based on our research, it is suggested that the surgeon should pay more attention to taking measures to reduce the degree of dental anxiety when such patients are admitted to the dental clinic, and the patients should take pain killing drugs when necessary after surgery.

This prospective study had several limitations. The sample size included in this study was too small; The hospital charged a higher fees for the third molar extraction, so the number of patients from rural areas in the surrounding towns was relatively small; (3) there was no breakdown for follow-up period, such as every four hours to evaluate the patient's pain perception; The patient's level of dental anxiety may be affected by other factors, such as the presentation of the postoperative instructions.

DA influenced the dentistry development. In the past few decades, the technology of dentistry treatment and equipment has been continuously improved, but the degree of DA was still high (24). DA disorders were not only related to patients rejecting dental treatment, affecting general health, and interfering with sleep, but also related to social interaction and work performance. Therefore, DA was a complicated problem that clinical dentists had to solve. From this study, we can get a general understanding of some influencing factors of patients with DA. For patients with DA, we can provide corresponding prevention and treatment measures, and use a variety of methods to jointly implement the treatment. We can strengthen the oral health education of patients, maintain a clean and tidy medical environment, and strengthen communication between doctors and patients during the process of receiving patients, to avoid pain and distract patients as much as possible.

## Conclusions

For patients (females, poor oral hygiene and no teeth extraction experience), surgeon should pay more attention to DA of such patients and take measures to reduce the anxiety of patients when removing the third molars. Furthermore, surgeon could recommend more effective pain killing drugs to improve post-operative pain in such patients.

## References

[1] Cui QY, Chen SY, Fu S, Zhang CB, Li M (2018). Survey and analysis of tooth extraction anxiety of dental patients. Hua Xi Kou Qiang Yi Xue Za Zhi.

[2] Seligman LD, Hovey JD, Chacon K, Ollendick TH (2017). Dental anxiety: An understudied problem in youth. Clin Psychol Rev.

[3] Asl AN, Shokravi M, Jamali Z, Shirazi S (2017). Barriers and Drawbacks of the Assessment of Dental Fear, Dental Anxiety and Dental Phobia in Children: A Critical Literature Review. J Clin Pediatr Dent.

[4] Sharma A, Pant R, Priyadarshi S, Agarwal N, Tripathi S, Chaudhary M (2019). Cardiovascular Changes Due to Dental Anxiety During Local Anesthesia Injection for Extraction. J Maxillofac Oral Surg.

[5] Montero J, Gomez-Polo C (2017). Personality traits and dental anxiety in self-reported bruxism. A cross-sectional study. J Dent.

[6] Reyes-Gilabert E, Luque-Romero LG, Bejarano-Avila G, Garcia-Palma A, Rollon-Mayordomo A, Infante-Cossio P (2017). Assessment of pre and postoperative anxiety in patients undergoing ambulatory oral surgery in primary care. Med Oral Patol Oral Cir Bucal.

[7] Lago-Mendez L, Diniz-Freitas M, Senra-Rivera C, Seoane-Pesqueira G, Gandara-Rey JM, Garcia-Garcia A (2006). Dental anxiety before removal of a third molar and association with general trait anxiety. J Oral Maxillofac Surg.

[8] Tarazona B, Tarazona-Alvarez P, Penarrocha-Oltra D, Rojo-Moreno J, Penarrocha-Diago M (2015). Anxiety before extraction of impacted lower third molars. Med Oral Patol Oral Cir Bucal.

[9] Maggirias J, Locker D (2002). Psychological factors and perceptions of pain associated with dental treatment. Community Dent Oral Epidemiol.

[10] Stouthard ME, Hoogstraten J (1990). Prevalence of dental anxiety in The Netherlands. Community Dent Oral Epidemiol.

[11] Boyle CA, Newton T, Milgrom P (2009). Who is referred for sedation for dentistry and why?. Br Dent J.

[12] Smith TA, Heaton LJ (2003). Fear of dental care: are we making any progress?. J Am Dent Assoc.

[13] Vermaire JH, de Jongh A, Aartman IH (2008). Dental anxiety and quality of life: the effect of dental treatment. Community Dent Oral Epidemiol.

[14] Tarazona-Alvarez P, Pellicer-Chover H, Tarazona-Alvarez B, Penarrocha-Oltra D, Penarrocha-Diago M (2019). Hemodynamic variations and anxiety during the surgical extraction of impacted lower third molars. J Clin Exp Dent.

[15] Humphris GM, Dyer TA, Robinson PG (2009). The modified dental anxiety scale: UK general public population norms in 2008 with further psychometrics and effects of age. Bmc Oral Health.

[16] Chen YW, Lee CT, Hum L, Chuang SK (2017). Effect of flap design on periodontal healing after impacted third molar extraction: a systematic review and meta-analysis. Int J Oral Maxillofac Surg.

[17] Malvania EA, Ajithkrishnan CG (2011). Prevalence and socio-demographic correlates of dental anxiety among a group of adult patients attending a dental institution in Vadodara city, Gujarat, India. Indian J Dent Res.

[18] Heft MW, Meng X, Bradley MM, Lang PJ (2007). Gender differences in reported dental fear and fear of dental pain. Community Dent Oral Epidemiol.

[19] Lago-Méndez L, Diniz M, Senra C, Seoane G, Gándara J, García A (2006). Dental anxiety before removal of a third molar and association with general trait anxiety. J Oral Maxillofac Surg.

[20] de Jongh A, Stouthard ME, Hoogstraten J (1991). Sex differences in dental anxiety. Ned Tijdschr Tandheelkd.

[21] Kanegane K, Penha SS, Munhoz CD, Rocha RG (2009). Dental anxiety and salivary cortisol levels before urgent dental care. J Oral Sci.

[22] Reyes-Gilabert E, Luque-Romero LG, Bejarano-Avila G, Garcia-Palma A, Rollon-Mayordomo A, Infante-Cossio P (2017). Assessment of pre and postoperative anxiety in patients undergoing ambulatory oral surgery in primary care. Med Oral Patol Oral Cir Bucal.

[23] Torres-Lagares D, Recio-Lora C, Castillo-Dali G, Ruiz-de-Leon-Hernandez G, Hita-Iglesias P, Serrera-Figallo MA (2014). Influence of state anxiety and trate anxiety in postoperative in oral surgery. Med Oral Patol Oral Cir Bucal.

[24] Smith TA, Heaton LJ (2003). Fear of dental care: are we making any progress?. J Am Dent Assoc.

